# Genetic Studies of Bone Diseases: Evidence for Involvement of DNA Damage Response Proteins in Bone Remodeling

**Published:** 2007-12

**Authors:** Xueying Wang, Baojie Li

**Affiliations:** *The Institute of Molecular and Cell Biology, Singapore*

**Keywords:** p53, Atm, c-Abl, osterix, osteoblast, osteoporosis

## Abstract

Bone remodeling is carried out by bone marrow mesenchymal stem cell derived osteoblasts, which form the bones, and hematopoeitic stem cell derived osteoclasts, which absorb the bones. Their actions are coordinated in two ways: osteoblasts and their precursors synthesize and secrete cytokines such as RANKL and M-CSF to regulate osteoclastogenesis; bone resorption releases matrix associated TGF-β and BMPs to stimulate bone formation at the same sites. Recent studies on transgenic mouse models revealed that several proteins involved in the DNA damage response play important roles in bone remodeling. DNA damage response is triggered by double stranded DNA breaks, single stranded DNA breaks as well as other types of lesions, which recruit and activate Ser/Thr kinases such as Atm to the damaged sites, where Atm activates p53 to promote apoptosis, cell cycle arrest, and DNA repair. Atm also activates c-Abl, a non-receptor tyrosine kinase, to promote apoptosis. Studies from our and other laboratories have shown that c-Abl and Atm positively regulate osteoblast differentiation and bone formation and mice deficient for either of them show osteoporosis, whereas p53 negatively regulates osteoblast proliferation/differentiation and bone formation and the knockout mouse shows osteosclerosis. These three proteins have osteoblast autonomous effect without directly affecting osteoclast differentiation or resorption activity. Furthermore, they appear to regulate osteoblast differentiation through controlling the expression of osterix, an osteoblast specific transcription factor essential for osteoblast differentiation. These results establish a functional link between osteoblast differentiation and DNA damage response.

## INTRODUCTION

### Bone development and bone-related diseases

The skeleton is made up of connective tissues and serves a few functions in mammals. It protects the internal organs and provides the environment for hematopoiesis. Moreover, it is also the site for calcium and phosphate storage. There are two types of bones: trabecular bones and cortical bones. While the former is formed by endochondral ossification using cartilage as a model, the latter is formed by intramembranous ossification. The trabecular bone mainly provides the niche for blood cell formation, while the cortical bone provides the strength (1, 2).

The skeleton experiences several phases of growth. Before the teenage years, the growth of the bones is slow. Entering puberty, under the influence of growth hormone and steroid hormones, bone formation surges and outpaces bone resorption, leading to marked bone growth in terms of bone size, bone mass and bone density. A few years post puberty, the bone reaches its peak mass (3). Then bone formation and resorption are kept at a constant rate and the bone mass does not change much until pre menopause. At menopause, bone resorption outstrips bone formation, leading to bone loss due to steroid hormone shortage (4). Osteoporosis is a bone related disorder with characteristics of reduced bone mass/density, deterioration of microstructure, and increased fracture risk. It affects more than 200 million people worldwide and causes morbidity, mortality, and a huge economic burden. It can be classified into senile osteoporosis, postmenopausal osteoporosis, and other pathological types such as glucocorticoid induced osteoporosis (2).

Postmenopausal osteoporosis is mainly caused by steroid hormone deficiency. It is more obvious in women than in men. This is because women lose estrogen at a faster rate than men lose testosterone. Steroid hormone deficiency results in enhanced osteoclastogenesis and increased osteoclast resorption activity, causing net bone loss (5, 6). On the other hand, senile osteoporosis is mainly caused by reduced bone formation due to a decrease in the number of osteoblasts, the activity of osteoblasts, or both, and affects both aged men and women (2). Opposite to osteoporosis is osteosclerosis, featured by an increase in bone mass and density. It is a relatively rare disorder that is mainly caused by an increase in bone formation (2, 7). In addition, there is also osteopetrosis, a disorder that is similar to osteosclerosis. However, osteopetrosis is mainly caused by dysfunctional bone resorption (8). Two of the most studied osteopetrosis models are c-Src knockout mouse and M-CSF (macrophage colony stimulating factor) mutant mouse. The former has dysfunctional osteoclasts that fail to absorb bones while the latter has reduced number of osteoclasts (9-11).

### Osteoblast and bone formation

Bone formation is carried out by osteoblasts that can synthesize collagen and other matrix proteins, and mineralize the bone matrix. They are derived from bone marrow mesenchymal stem cells (MSC), which can also give rise to myoblasts, adipocytes, and chondrocytes (2). Maturation of MSCs to osteocytes is a multi-step process that requires both cell expansion and differentiation. At each step, the cells express specific markers. The most commonly used markers are alkaline phosphatase (ALP), a relatively early marker, osteocalcin, a medium stage marker, and bone nodule formation, a late marker. These markers can be used to assess the progression of osteoblast differentiation.

Osteoblast differentiation is controlled by osteoblast specific transcription factors (12, 13). Runx2, a runt domain containing protein, promotes MSC differentiation not only to osteoblasts but also to chondrocytes (14). Osterix, a zinc finger protein, acts at later steps (15). Both Runx2 and osterix are essential for osteoblast differentiation and bone mineralization, as seen in Runx2 or osterix deficient mice, whereby there are no mature osteoblasts or calcified bones (15-17). Furthermore, overexpression of Runx2 or osterix in non-osteoblast cells induces the expression of osteoblast markers, suggesting that they are sufficient in promoting osteoblast differentiation (14, 15). In addition, some transcription factors, though expressed in other cell types as well, play crucial roles in bone development or remodeling. Among them are Sox9, Dlx5, and Atf4. Mice deficient in each of them also show bone related defects (18-20). It is conceivable that controlling the expression of transcription factors such as Runx2 or osterix would be an important measure in regulating osteoblast differentiation and bone remodeling.

Osteoblast differentiation is also influenced by extracellular stimuli. Among the best studied are BMPs, Lpr5/Wnt, and mechanical loading (4). While the signaling pathways that are triggered by BMPs and Lpr5/Wnt are established, the molecular mechanisms by which these growth factors control osteoblast differentiation are less well understood. It is possible that these signaling molecules may directly or indirectly regulate the expression of the above mentioned transcription factors such as Runx2 and osterix.

### Osteoclast and bone resorption

Osteoclasts are very large multinucleated cells that grow up to 100 μm in diameter and are responsible for bone resorption. They exist in varying shapes, depending on the resorption activity of the cells. They also contain numerous pleomorphic mitochondria, a variety of vesicular structures, and a ruffled edge, where active resorption takes place. These cells also help in the maintenance of mineral homeostasis of the extracellular fluid (21, 22). The rate of bone resorption could be regulated by either the number of osteoclasts or the resorptive activity of mature osteoclasts. Excessive activity of osteoclasts, which is common in postmenopausal women, causes osteoporosis due to rapid bone loss, while reduced activity of osteoclasts causes osteopetrosis due to lesser bone resorption (23, 24).

Osteoclasts are derived from hematopoietic stem cells (HSCs) in the bone marrow (25, 26). Their formation requires two steps, the first step of which is the differentiation of HSCs to osteoclast progenitors, a common precursor for monocytes and macrophages as well. The second step is the differentiation of mature osteoclasts from these progenitors, whereby multinucleated osteoclasts form from fusion of precursor cells on the bone surface.

Many factors including cytokines, hormones and even extracellular matrix molecules like osteopontin and osteocalcin, play a role in osteoclastogenesis. Positive regulators include various cytokines and hormones such as IL-1β, 6, 11, 17, GM-CSF, M-CSF, TNF-α and PTH (26, 27). Negative regulators include factors like INF-γ, TGF-β, pharmacological inhibitors like SERMs and bisphosphates, and many others (27). M-CSF (also known as colony-stimulating factor 1 (CSF-1)) is a lineage-specific growth factor for mononuclear phagocytes. It can be synthesized by mesenchymal cells. Osteoclast precursors have on their cellular surface c-Fms, receptor for M-CSF. M-CSF is essential for recruitment, differentiation, migration, activity and survival of osteoclasts, as well as for proliferation and activity of monocytes and macrophages (26, 28). In the op/op mouse model, which is deficient in M-CSF due to an extra thymidine insertion in the coding region of the *M-CSF* gene, there are not only low numbers of macrophages and monocytes, but also an almost practical absence of osteoclasts. As a result, the mouse develops osteopetrosis (11).

Osteoclastogenesis also requires osteoclast differentiation factor (ODF, also called RANKL/TRANCE/OPGL). Osteoclast precursors express RANK, a receptor for RANKL on the cell surface. RANKL thus promotes osteoclast formation, activation and survival upon engagement with the receptor (25). M-CSF is known to induce RANK in osteoclast precursors. OPG (OCIF) is the naturally occurring decoy receptor and inhibitor of RANKL. It functions as a paracrine inhibitor of osteoclasts, curbing both their production and activity. The balance between RANKL and OPG determines the pace of osteoclastogenesis and bone resorption.

Among many transcription factors that control osteoclastogenesis, PU.1 (also known as Spi-1) is a member of the Ets family that has been implicated in a wide range of physiological and pathological processes (29). PU.1 is only expressed in hematopoietic cells, predominantly in myeloid and B cells, but not in T cells. It is also a proto-oncogene and is implicated in murine acute erythroleukemia (29). Mice with targeted disruption of PU.1 gene are osteopetrotic, in addition to having multiple hematopoietic abnormalities such as a complete lack of myeloid or lymphoid precursors, and the absence of B cells, dendritic cells and macrophages (30-32).

Tartrate resistant acid phosphatase (TRAP) is secreted in substantial amount by osteoclasts. It is the most common biochemical marker for osteoclast differentiation assessment. Another highly specific phenotypic marker of osteoclasts is cathepsin K, whose mRNA levels could be assessed by RT-PCR or real-time PCR. Osteoclasts could also be characterized by its multi-nucleation feature. Formation of resorption pits is used to assess the bone resorption function of osteoclasts *in vitro*. An *in vivo* marker for bone resorption is the urine level of collagen-breakdown product, deoxypyridinoline cross-links.

### Coupling between osteoblasts and osteoclasts

An indication of coupling between osteoblastogenesis and osteoclastogenesis comes from studies of bone formation and resorption markers (27). Markers for both processes tend to follow the same pattern during bone remodeling. Their levels are high during childhood and teenage years, drop in adulthood and increase again after menopause. These findings have led to the concept that the two processes of bone formation and resorption are mechanistically coupled. The resorbed bone can be filled up by newly formed bones. Thus, osteoclastic bone resorption is always followed by osteoblastic bone formation and the two processes are coordinated. It is believed that insulin-like growth factors (IGFs) and TGF-β are growth factors released during resorption that stimulate bone formation (25, 33). Great attempts have been taken to identify other coupling factors between osteoblasts and osteoclasts bone remodeling.

Increasing evidence suggests that there exists another layer of coupling between osteoblast and osteoclast. For example, osteoclastogenesis and bone resorption is known to be controlled by PTH. Yet, PTH does not have a direct effect on osteoclasts as pure osteoclast cultures do not respond to PTH stimulation. Osteoclastic bone resorption responds to PTH in the presence of other bone marrow derived cells such as osteoblasts, indicating that the effect of PTH on osteoclastogenesis is mediated by osteoblasts (34). It is now known that active osteoclast maturation requires the presence of coupling factors from other bone-marrow derived cells including osteoblasts, monocytes and lymphocytes (25, 35). Increasing amount of evidence indicate that osteoblasts and their progenitors can synthesize and present growth factors to osteoclasts to regulate osteoclastogenesis. For example, RANKL and M-CSF could be synthesized by osteoblastic precursors to stimulate osteoclastogenesis. The proximity between the osteoblastic lineage and hematopoietic cells is required for RANKL and M-CSF to bind to their cognate receptors (RANK and c-Fms respectively), which are expressed on the surface of monocytes and macrophages. RANKL and M-CSF engagements then stimulate proliferation and differentiation of osteoclasts. Negative regulator OPG could also be synthesized and secreted by osteoblasts and their precursors. In addition, osteocytes are known to stimulate osteoclast resorptive activity and are possibly involved in bone resorption upon PTH exposure (36).

### DNA damage and tumorigenesis

DNA is the blueprint for operation of each cell. Damaging it not only disturbs the repository of hereditary information that determines our very phenotypic appearance, but also causes cancer and aging. DNA damage can be caused by exogenous agents such as ionizing radiation (IR) and ultraviolet (UV) light exposure, and endogenous factors such as reactive oxygen species that are generated by mitochondria in the process of ATP production. Many types of DNA damage are generated depending upon the genotoxic stress encountered by the cell. There are three major types of DNA lesions: double stranded DNA breaks (DSBs), single-stranded DNA breaks (SSBs) and base/nucleotide modifications (37).

Depending on the types and the severity of DNA lesions, cells respond to DNA damage in different ways. When cells sense that the extent of the damage is not severe, cell-cycle checkpoints are activated and cells will stop dividing (38). During the pause, DNA repair enzymes start to fix the damages and the cells will be rescued. On the other hand, when cells sense that the severity of the damage is beyond repair, it may activate apoptotic signaling cascades, resulting in the elimination of these cells.

DNA damage is involved in tumorigenesis. Many of the proteins involved in DNA damage response, including sensors, signaling molecules, effector molecules and DNA repair proteins, are found to be involved in tumorigenesis (39). Furthermore, it was recently reported that in many cell types, the conversion from normal to cancer cells is accompanied by activation of the DNA damage response (40, 41). The function for this activation is to inhibit cell proliferation or to induce apoptosis. As a result, cells with mutations in proteins involved in DNA damage response are selected and become cancerous. Thus, DNA damage response is a protective mechanism against cancer development (42).

### Atm and DNA damage response

**Figure 1:**
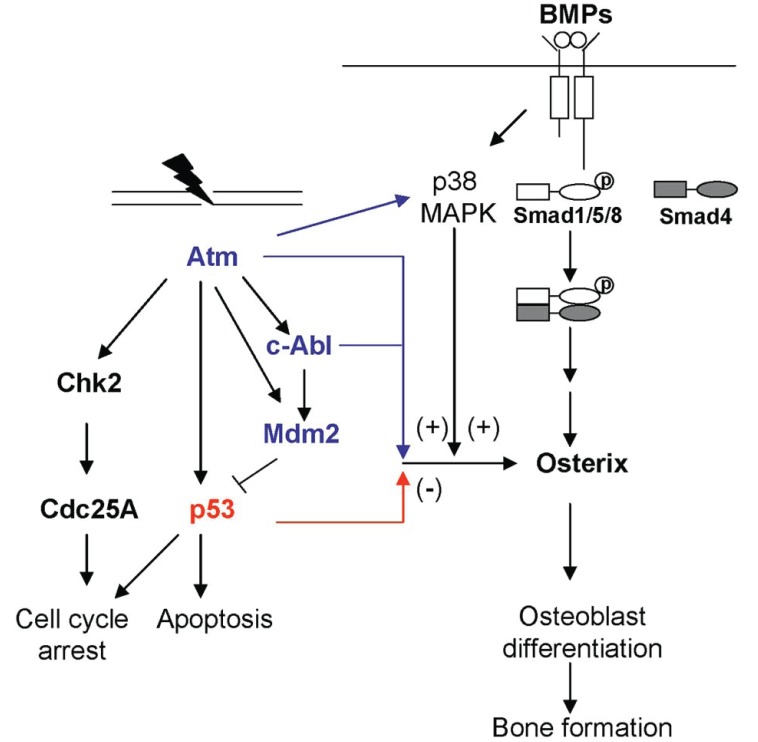
A diagram showing the crosstalk between the DNA damage response pathway and BMP-controlled osterix expression and osteoblast differentiation.

DNA damage activates multiple signaling cascades, which assist in decision-making to affect a particular cellular response. At the center of these damage triggered signaling network are phosphoinositide-3-kinase-like kinases (PIKKs) that include members like DNA-PKcs, Ataxia Telangiectasia Mutated (Atm) and Ataxia Telangiectasia and Rad3-related (Atr) (43-45). Despite their homology to lipid kinases of the PI-3K family, PIKK family members seem to be exclusive serine/threonine (Ser/Thr) kinases. Atm responds mainly to DSB, while Atr is activated by ssDNA and stalled DNA replication forks. Atr is also the main sensor activated under UV induced damage. DNA synthesis replication blockade agents like hydroxyurea and aphidicolin activate only Atr as well (46). Atm was initially identified by positional cloning and was mapped on chromosome 11. The gene is organized into 66 exons. Atm is mainly a nuclear protein in the form of dimers or multimeric complexes, with the kinase domain of one molecule bound to the other through intermolecular interaction (47, 48). However, upon exposure to adriamycin or IR induced DSBs, there is an alteration in the chromatin structures. This induces rapid intermolecular phosphorylation of Atm dimers on the residue Ser1981. The auto-phosphorylation in the FAT domain of Atm leads to the dissociation of the previously inert dimer complex. As a result, active Atm monomers are then free to migrate to sites of DSBs and phosphorylate substrates such as p53 and cell cycle checkpoint protein kinase 2 (Chk2) (Fig. 1) (43).

Atm interacts with and phosphorylates a broad network of proteins, including Chk2, tumor suppressors p53 and Brca1, DNA repair factors such as RAD50, and other signaling molecules such as c-Abl (Abelson Tyrosine Kinase) (49). Atm is recruited to and activated at sites of DNA DSBs, where it initiates cell signaling events to induce cell cycle arrest at the G1/S mainly through phosphorylation of p53 at Ser15 and up-regulation of p21 (43). Moreover, Atm also phosphorylates and activates Chk2, which subsequently phosphorylates p53 at Ser 20 in humans (Ser 23 in mouse), stabilizing the p53 protein. Activated Chk2 is also involved in cell cycle checkpoint at G2/M phase. Under severe DNA damage, Atm activated p53 could also lead to apoptosis. Meanwhile, Atm–mediated phosphorylation of other DNA damage response proteins like Brca1, Nbs1 and Smc1 leads to a variety of effects on DNA repair, cell-cycle progression and apoptosis (50).

Mutations in Atm in humans cause a disorder called Ataxia-Telangiectasia (A-T), an autosomal recessive neurodegenerative disease (51). Mutations have been found in all parts of the *Atm* gene in the genomic DNA extracted from A-T patients. A-T patients are at increased risk of cancer. They also show increased susceptibility to infections due to immunodeficiency, and symptoms of premature aging, most of which are manifested in the Atm-/- mice (52, 53). Furthermore, Atm is essential in the self-renewal of HSCs. It protects the HSCs pool from oxidative stress (54). At the cellular level, cells from A-T patients manifest hypersensitivity to IR and other agents that induce DSBs. A similar acute radiosensitivity is also seen in A-T patients (44).

### c-Abl in DNA damage response

c-Abl was identified as the cellular homologue of v-Abl (viral-Abl), an Abelson Murine Leukemia Viral oncogene (A-MuLV) (55, 56). The gene *ABL1* encodes a 150 kDa tyrosine kinase. The protein has attracted much attention because of its structural alikeness to BCR-ABL, the leading cause of human Chronic Myeloid Leukemia (CML), and also because of its essential role in mouse development (57, 58). BCR-ABL is responsible for approximately 90% of adult CML, 20% of adult ALL (acute lymphoblastic leukemia) and 2% of adult AML (acute myeloid leukemia) (59).

c-Abl is a member of the Sarcoma (Src) family of non-receptor tyrosine kinases (NRTK), sharing common features in domain sequences and organization with Src. It catalyzes tyrosine phosphorylation on target molecules (60). Active c-Abl has been implicated in tumorigenesis and in many cellular processes including differentiation, cell division, adhesion and DNA damage-induced apoptosis (61).

Numerous studies indicate that c-Abl plays an important role in DNA damage response (62). DSBs were reported to activate c-Abl in an Atm-dependent manner (63, 64). Activated Atm was found to interact with and phosphorylate c-Abl, leading to its activation. Activated c-Abl could then stabilize both p53 and p73 to promote apoptosis (61, 65). Moreover, c-Abl has been reported to interact with many other proteins involved in DNA damage response. These include p53, Wrn and Brca1 (66, 67). Yet the role for c-Abl in DNA damage induced cell cycle control and DNA repair is still controversial.

### p53 in DNA damage response

p53 was identified in 1979 as a 53 kDa protein (hence the name) that interacts with the Simian SV40 large T antigen. *p53* was initially thought as an oncogene that promotes cell proliferation. However, it was later found that the oncogenic forms are the mutant p53 molecules. The wild type *p53* is in fact a tumor suppressor gene that acts as a gatekeeper to suppress tumor growth and is capable of inhibiting the transformation of many cell types *in vitro* (68, 69). p53 knockout mice were then generated and found to be prone to cancer development (70). Later, the importance of p53 in cancer progression is illustrated by the fact that p53 is mutated in over 50% of all human primary tumors, making it a best-studied tumor suppressor (69). It responds to a variety of endogenous and exogenous cellular signals, and it triggers diverse biological responses such as DNA repair, cellular senescence and cell-cycle checkpoints. In recent years, the roles of p53 in cellular differentiation and development have also been studied (71).

p53 has the ability to prevent cancer development by inducing programmed cell death, cell cycle arrest, terminal differentiation, or senescence. Hence, in cells without functional *p53*, the increased predisposition to tumor development is due to the accumulation of genetic alterations after damage (72). p53 is a transcription activator for some target genes. It can bind to the specific DNA sequences to activate transcription. p53 executes its various functions by regulating different sets of target genes. For example, in response to DNA damage, accumulated and activated p53 could induce the expression of Bax, Puma, and Noxa to promote apoptosis (Fig. 1). It could also induce the expression of p21 and Gadd45 to activate cell cycle checkpoints (73). In addition, p53 can also act as a transcription repressor for some target genes, in a DNA binding dependent or independent manner (74).

### DNA damage response proteins in bone remodeling

With the strategy of reverse genetics, the possible roles for Atm, c-Abl, and p53 in bone remodeling have been studied by ourselves and other groups. Analysis of the mouse bone mass and density, bone formation and resorption rates, and proliferation and differentiation of osteoblasts and osteoclasts established three mouse models for bone-related diseases: Atm-/- mice as a model for post-menopausal osteoporosis, c-Abl-/- as a model for senile osteoporosis and p53-/- mice as model for osteosclerosis. Further studies with biochemical and cell biological measures provided some insight into the molecular mechanisms by which c-Abl, Atm, and p53 regulate osteoblast differentiation and proliferation (75-80).

### c-Abl

c-Abl plays an important role in mouse development. More than 50% of the mice deficient for c-Abl show neonatal lethality (died 1 to 2 weeks after birth). The rest of the mice can survive up to a few months. They are runted, sterile and display morphological abnormalities such as foreshortened crania. These mice are also susceptible to infections, probably due to T and B cell lymphopenia as well as thymic and splenic atrophies (81, 82). In addition, mice deficient for c-Abl and its only paralogue, ARG (Abl related gene), are embryonic lethal with defective neural tube closure and massive apoptosis (83).

A bone related phenotype was observed when bone marrow pre-B and pro-B cells were isolated: the femur bones were found to be very fragile. Further studies demonstrated that c-Abl plays an important role in bone remodeling, consistent with the observation that high levels of c-Abl are expressed in hyaline cartilage in adults, in bone tissue of newborn mice, in bone-forming osteoblasts, and also in associated neo-vasculature at sites of endochondral ossification in the mouse fetus (84, 85). Dual X-ray absorptometry and histomorphometry analysis show that c-Abl deficient mice have reduced bone mineral density, thinner cortical bones, reduced trabecular bone volume, and reduced bone mass, which are characteristics of osteoporosis. Examination of the bone-resorbing osteoclasts revealed no significant defects in their number and function. Calcein labeling experiments demonstrated that c-Abl deficient mice have markedly reduced bone formation. Moreover, c-Abl deficient osteoblasts show defects in their differentiation and maturation. Osteoblasts isolated from both the bone marrow cells and purer calvarial populations expressed lesser markers such as ALP, osteocalcin and mineral deposition (80).

We also found down-regulation of osterix in c-Abl-/- calvarial osteoblasts, indicating that the positive role of c-Abl in osteoblast differentiation could be mediated by osterix (75). In summary, it was found that i) c-Abl exerts a positive and cell-autonomous role in osteoblast differentiation and bone formation without affecting osteoclast and bone resorption; ii) c-Abl knockout mice might serve as a mouse model for senile osteoporosis, which is mainly caused by a reduction in bone formation due to a decline in the number and the function of osteoblasts; iii) c-Abl is likely to regulate osteoblast differentiation through modulating the expression of osterix, a transcription factor essential for osteoblast differentiation and whose levels control the progression of the differentiation process; iv) it also provides a clue to the function of c-Abl in bone remodeling, as osterix is under the control of BMPs.

### Atm

Since c-Abl deficient mice are defective in bone remodeling, a possible role in bone remodeling for Atm, a c-Abl interacting protein and an upstream kinase essential for c-Abl activation in DNA damage response, was also tested (64, 67). Atm is not absolutely required for mouse development as homozygote null mice look normal although they develop lymphomas at 5-6 month of age and display A-T like phenotypes such as neurodegeneration, radiosensitivity, infertility, immune deficiency, and premature aging (53).

Atm knockout mouse show reduced bone mass and bone density, characteristics of an osteoporotic phenotype (78, 79). This is caused by both decreased bone formation and increased bone resorption. While the decrease in bone formation in Atm-/- mice is accompanied by compromised osteoblast differentiation, the number of osteoblasts and the osteoblast surface on bone sections are comparable to that of wild type mice (78). Moreover, Atm deficiency has no effect on stem cell renewal for osteoblast lineage cells, which is supported by the same number of ALP-positive-CFU colonies in the osteoprogenitor population from Atm-/- marrow and control littermates. However, another study suggests that Atm-/- mice develop osteopenia due to a defect in osteoblast stem cell renewal (79). The discrepancy can be caused by differences in the genetic background and in the ages of mice used in studies carried out by different laboratories.

Atm deficiency leads to defective osteoblast differentiation and this involves osterix. The levels of osterix, but not that of Runx2, Atf4 and other transcriptional factors, are markedly reduced in primary Atm-/- calvarial osteoblasts. Atm deficiency also compromised BMP-induced osterix expression at both mRNA and protein levels, suggesting a possible role for Atm in the BMP signaling (78). It is also possible that Atm regulates osteoblast differentiation through the IGF1 signaling or the p38 MAPK signaling pathway (79). This is because Atm deficiency compromises the expression of IGF1 receptor and it is well known that IGF1 signaling pathway plays an important role in bone remodeling (86). Furthermore, p38 MAPK has been shown to be required for osteoblast differentiation as well, possibly via controlling the expression of osterix (Wang *et al*, unpublished results). These results suggest that Atm may affect osteoblast differentiation by modulating osterix expression.

Atm-/- mice also show increased bone resorption, which is reflected by an increase in the number of osteoclasts, the osteoclast surface, and urine deoxypyridinoline cross-links excretion. However, neither the differentiation of Atm-/- bone marrow monocytes to osteoclasts nor the bone resorption activity of these osteoclasts is affected by Atm deficiency. These results indicate that Atm has no cell-autonomous role on osteoclastogenesis or resorption (78). Since Atm-/- mice are known to have defective gametogenesis and infertility (53), traits that are also true for A-T patients, the levels of steroid hormones in these mutant mice were determined. Both male and female Atm-/- mice show severe gonad atrophy, accompanied by reduced levels of serum/urine testosterone in males and reduced levels of serum/urine estrogen in females (78). Ovariectomy, orchidectomy and the consequent abrogation of gonad steroid hormones are well-known to enhance both osteoclastogenesis and bone resorption, leading to osteoporosis (87). Hypogonadism can be of central origin, in which pituitary secretes less gonodotrophic hormone FSH (follicle stimulating hormone) and LH (Leutinizing hormone), which are essential for normal gonad development and for production of testosterone and estradiol. Alternatively, hypogonadism can be of peripheral origin, in which the defect is due to the development of gonad itself. Serum FSH and LH levels were determined and FSH levels show a marked up-regulation while LH levels do not change much. This finding reflects a hypogonadism of peripheral origin, in which the elevation of FSH and/or LH is a result of feedback regulation from steroid hormone deficiency (88, 89). Later, FSH was shown to have a direct effect on bone loss by binding to receptors on osteoclasts to induce resorption (90).

These studies on Atm-/- mice contribute to the understanding of bone remodeling in several aspects. First, the female Atm-/- mice represent a model for post-menopausal osteoporosis, which could offer insight into the therapeutic measures against this disorder. Secondly, the link between Atm deficiency and steroid hormone shortage was established, which might be responsible for some of the other symptoms affecting A-T patients. Thirdly, genetic evidence supports a role for Atm, by affecting DNA recombination during meiosis, to influence bone remodeling. Lastly, these studies also imply that Atm might participate in the BMP signaling pathways.

### p53

The fact that both Atm and c-Abl play important roles in bone remodeling promoted us to examine a possible role for p53 in the same process. In addition, previous studies also suggest that p53 might play a role in bone remodeling. First, 17-day-old embryos deficient in p53 showed slight skeletal defect characterized by reduced embryonic bone length and width. This is probably due to a delay in the maturation of the cartilage (91). Secondly, *in vitro* administration of estrogen increases p53 protein transactivity, together with enhanced osteoblastic-specific markers, ALP and osteocalcin. This p53 elevation is associated with cell cycle arrest rather than apoptosis (92, 93). Thirdly, mechanical unloading of mice by tail suspension was found to result in a reduction of trabecular bone volume and bone formation rate due to defective osteoblast differentiation in wild type, but not in p53-/- mice (94).

Dual x-ray absorptometry and histomorphometry analysis of p53-/- vs control littermates revealed an osteosclerotic phenotype in the mutant mice. p53-/- mice show slightly increased bone mineral density and increased bone volume (75). Surprisingly, these mutant mice show increased bone formation and increased bone resorption. As the overall result is osteosclerosis, increased bone formation has to be dominant. Thus p53-/- mice is a model of increased remodeling with an osteosclerotic phenotype.

Increased bone formation in p53-/- mice is accompanied by an increase in the number of osteoblasts and an enhancement of osteoblast differentiation, two cellular events that are generally exclusive. Calvarial osteoblasts show increased doubling rates that can be restored to normal by expressing p21, the target gene of p53 and a crucial player in determining the growth rate. On the other hand, enhanced differentiation is not affected by the expression of p21 (75). These results suggest that p53 controls these two processes with distinct mechanisms. Further studies indicated that enhanced differentiation is mediated by elevated expression of osterix, and that p53 can directly suppress osterix promoter activity in a DNA binding independent manner. Overexpression of osterix in normal osteoblasts could mimic the differentiation phenotype of p53-/- cells. Knocking down of osterix in p53-/- osteoblasts with siRNA slowed down differentiation. These results suggest that elevated expression of osterix mediates the effect of p53 deficiency on osteoblast differentiation.

It appears that c-Abl and p53 play opposite roles in osteoblast differentiation. Previous studies also suggest a physical and functional interaction between these two proteins in DNA damage response (95). A functional relationship in osteoblast differentiation and bone development between c-Abl and p53 was also tested. c-Abl-/- and p53-/- mice were crossed to generate double knockout mice. Unfortunately, the double knockout mice can not be obtained due to embryonic or perinatal lethality. However, osteoblasts deficient for both c-Abl and p53 could be isolated from 20 day embryos. Differentiation assays revealed that the double knockout osteoblasts behaved just like p53-/- cells, suggesting that p53 acts downstream of c-Abl in osteoblast differentiation (75).

p53 deficient mice also serve as a model for coupling between osteoblastogenesis and osteoclastogenesis. p53-/- mice show enhanced bone resorption, manifested by an increase in the number of osteoclasts, the bone resorption surface and the secretion of deoxypyridinoline cross-links. Like Atm, p53 has no cell-autonomous effect on osteoclast differentiation from monocytes or the resorption activity of the monocyte derived osteoclasts. On the other hand, p53-/- osteoblasts acquired enhanced osteoclastogenic activity as demonstrated in a co-culture experiment. More interestingly, the enhanced osteoclastogenesis is likely caused by an up-regulation of M-CSF, which is induced by ectopic expression of osterix. The osteoblast-supported osteoclastogenesis data also help to explain why osteosclerotic models like p53-/- mice only show moderate increase in bone mass while they have markedly enhanced bone formation. These findings indicate that p53 indeed plays a very important role in skeletal formation: it directly inhibits bone formation and indirectly inhibits bone resorption (75).

This conclusion was further supported by another study. Mdm-2, a negative regulator of p53 that is expressed at early stages of mouse development, was specifically deleted from osteoblasts. These mutant mice were found to have reduced osteoblast proliferation and differentiation, in association with increased p53 transactivity. In contrast, osteoprogenitor cells from mice deficient for p53 have the exact opposite phenotype with an overall increased bone mass in addition to expected development of osteosarcomas. Moreover, p53 deficiency was able to rescue the bone defect of Mdm2-/- mice (76). These results together have placed p53 as an important safeguard in osteoblast differentiation in addition to cell proliferation and cancer development. Hence, p53-/- mice can be used as an osteosclerotic animal model, which may allow us to comprehend factors coupling bone formation and bone resorption during bone remodeling.

### Common features of c-Abl, Atm and p53 in regulating bone remodeling

We have described the roles of c-Abl, Atm, and p53 in bone remodeling, which are supported by *in vivo* data, and by cell biological and biochemical evidence. These studies not only established mouse models for bone-related diseases but also provided evidence that DNA damage response proteins have novel functions in development. Below, we summarize some of the common features of these mouse models.

### Cell autonomous effect on osteoblast differentiation

All three knockout mice showed bone related phenotypes, which can be attributable to altered function of osteoblast and bone formation, altered osteoclast function and bone resorption, or both. However, cell biological studies on the primary osteoblasts or their progenitors indicate that none of the three genes (c-Abl, Atm, and p53) have cell autonomous effects on osteoclastogenesis or the resorption activities of these osteoclasts. On the other hand, osteoblasts derived from these three deficient mice all show an alteration in differentiation, which is manifested by the altered expression of osteoblast specific markers and bone mineralization (75, 76, 78, 80). The question remains why c-Abl, Atm, and p53 specifically regulate the differentiation of mesenchymal derived osteoblast but not the HSC derived osteoclasts.

### Secondary effects on bone resorption

While c-Abl deficient mice do not show a significant defect in bone resorption, both Atm deficient and p53 deficient mice show an alteration in bone resorption (75, 78). Since neither Atm nor p53 has a direct effect on osteoclast differentiation or resorption, the observed change in bone resorption has to be a secondary effect. Further studies confirm this prediction. It was found that Atm deficient mice also show hypogonadism and reduced serum levels of steroid hormones, which is known to promote bone resorption. This hypogonadism is caused by a direct effect of Atm on gonad development. On the other hand, p53 deficient osteoblasts show enhanced osteoclastogenic activity compared to normal osteoclasts. Therefore, a p53 deficient mouse can be used to study coupling between bone formation and bone resorption.

### Converging at osterix expression

What are the molecular mechanisms by which these proteins regulate osteoblast differentiation and bone formation? While the details of the processes involved need further investigation, the available data provide some important clues. In osteoblasts deficient for each of the three genes, there is an alteration in the expression of osterix, which is positively related to the differentiation potential (15). This positive correlation is only specific to osterix but not to other transcription factors such as Runx2, Atf4, or Dlx5 (75, 78). For example, osteoblasts deficient in c-Abl or Atm show defective differentiation that is accompanied by reduced levels of osterix. On the other hand, p53 deficient osteoblasts show enhanced differentiation that is accompanied with increased levels of osterix (Fig. 1). In addition, inhibition of p38 MAPK impedes osteoblast differentiation as well as the expression of osterix (Wang X., unpublished results). Inhibition of Cox-2 also compromises osteoblast differentiation and reduces the expression of osterix. More importantly, we found that knocking down osterix in p53 deficient osteoblasts slowed down differentiation while overexpression of osterix render resistance to p38 MAPK inhibitor in regard to differentiation (Wang X., unpublished results). These functional studies, in conjunction with the fact that osterix is necessary and sufficient for osteoblast differentiation and *in vivo* bone calcification, suggest that osterix, at least partially, mediates the effect of these proteins on osteoblast differentiation and bone remodeling.

Osteirx is under the control of BMPs and IGFs (96, 97). BMPs are the driving force of osteoblast differentiation and bone formation *in vivo*. BMPs induce osterix transcription through the p38 MAPK pathway in addition to the Smad1/5/8 pathway (Fig. 1). The fact that c-Abl and Atm positively regulate osterix suggests that these two proteins might be involved in the BMP signaling pathway, either Smad1/5/8 or the p38 MAPK pathway. Further investigation will be needed to determine how c-Abl, a tyrosine kinase, and Atm, a Ser/Thr kinase, participate in the BMP signaling pathway.

### Perspectives

DNA damage response and repair are known to be associated with tumorigenesis (39). Loss of Atm predisposes the patients and the mice to lymphoma and leukemia as well as other types of cancers. Activated c-Abl forms, BCR-ABL and v-Abl, can transform lymphocytes and cause leukemia in humans and mice respectively (57). Loss of p53 function occurs in more than 50% of different types of primary tumors (69). In the mouse, loss of p53 leads to development of lymphoma as well as other types of tumors (70). This is mainly because Atm, c-Abl, and p53 are activated by DNA damage and their function is to trigger cell cycle checkpoints, apoptosis, and repair. Here we draw the conclusion that all three proteins are involved in osteoblast function, bone formation and bone remodeling, without a cell-autonomous effect on osteoclast and bone resorption. While it has been well accepted that oncogene products and tumor suppressors play important roles in mammal development, the question remains why these three proteins, which are involved in DNA damage response and tumorigenesis, specifically affect the differentiation of osteoblasts. Is there anything in common between DNA damage response and osteoblast differentiation?

A striking finding in recent years is that DNA damage response and repair proteins are associated with neuron degeneration and other neurological diseases (98). Atm deficiency causes ataxia (51). Nsb1, a sensor protein that recruits Atm to double stranded DNA breaks, is involved in development of microcephaly (99). Deficiency of Wrn, a helicase involved in DNA repair, leads to progressive neurodegeneration (100). Aprataxin and TDP1, both required for single stranded DNA repair, are involved in the development of spinocerebellar ataxia syndrome (98). While the connection between DNA damage response/repair and neurodegeneration has been well established, the molecular mechanisms behind this link are not clear.

One possible explanation for the link between DNA damage response and osteoblast differentiation is that this conserved signaling pathway controls expression of distinct sets of genes in different cell types. While in most cell types, Atm, c-Abl and p53 control DNA damage induced cell cycle progression and apoptosis by regulating the expression of p21 and Bax as well as other proteins; in osteoblast, these three proteins also regulate a lineage specific transcription factor osterix. Since osterix is not expressed in other cell types, the link of DNA damage proteins to differentiation is only observed in the osteoblasts. Notably, the action of Atm, c-Abl and p53 in osteoblast differentiation might not involve stress response. However, in response to DNA damage, the response to control cell cycle progression and apoptosis will be the dominant one, even though osterix levels could be altered. Indeed, osterix has been proposed as a tumor suppressor in development of osteosarcoma, the most common form of bone cancer and the second most frequent malignancy following retinoblastoma. Osteosarcoma is a primary bone cancer as it develops in growing bones, normally from osteoblasts of mesenchymal origin (101). Osterix was found to be down-regulated in osteosarcoma lines and ectopic expression of osterix inhibits proliferation of these cells (102). Further investigation will be needed to test whether osterix is a main mediator in DNA damage induced cell cycle arrest and/or apoptosis in osteoblast.
